# Interleukin-1 blockade attenuates white matter inflammation and oligodendrocyte loss after progressive systemic lipopolysaccharide exposure in near-term fetal sheep

**DOI:** 10.1186/s12974-021-02238-4

**Published:** 2021-08-31

**Authors:** Sharmony B. Kelly, Vanesa Stojanovska, Valerie A. Zahra, Alison Moxham, Suzanne L. Miller, Timothy J. M. Moss, Stuart B. Hooper, Marcel F. Nold, Claudia A. Nold-Petry, Justin M. Dean, Laura Bennet, Graeme R. Polglase, Alistair J. Gunn, Robert Galinsky

**Affiliations:** 1 The Ritchie Centre, Hudson Institute of Medical Research, 27-31 Wright street, Melbourne, Victoria 3168 Australia; 2 Department of Obstetrics and Gynaecology, Monash University, Melbourne, Victoria Australia; 3 Department of Paediatrics, Monash University, Melbourne, Victoria Australia; 4 Monash Newborn, Monash Children’s Hospital, Melbourne, Australia; 5 Department of Physiology, The University of Auckland, Auckland, New Zealand

**Keywords:** Inflammation, Brain, Interleukin-1

## Abstract

**Background:**

Increased systemic and tissue levels of interleukin (IL)-1β are associated with greater risk of impaired neurodevelopment after birth. In this study, we tested the hypothesis that systemic IL-1 receptor antagonist (Ra) administration would attenuate brain inflammation and injury in near-term fetal sheep exposed to lipopolysaccharide (LPS).

**Methods:**

Chronically instrumented near-term fetal sheep at 0.85 of gestation were randomly assigned to saline infusion (control, *n* = 9), repeated LPS infusions (0 h = 300 ng, 24 h = 600 ng, 48 h = 1200 ng, *n* = 8) or repeated LPS plus IL-1Ra infusions (13 mg/kg infused over 4 h) started 1 h after each LPS infusion (*n* = 9). Sheep were euthanized 4 days after starting infusions for histology.

**Results:**

LPS infusions increased circulating cytokines and were associated with electroencephalogram (EEG) suppression with transiently reduced mean arterial blood pressure, and increased carotid artery perfusion and fetal heart rate (*P* < 0.05 vs. control for all). In the periventricular and intragyral white matter, LPS-exposure increased IL-1β immunoreactivity, numbers of caspase 3+ cells and microglia, reduced astrocyte and olig-2+ oligodendrocyte survival but did not change numbers of mature CC1+ oligodendrocytes, myelin expression or numbers of neurons in the cortex and subcortical regions. IL-1Ra infusions reduced circulating cytokines and improved recovery of EEG activity and carotid artery perfusion. Histologically, IL-1Ra reduced microgliosis, IL-1β expression and caspase-3+ cells, and improved olig-2+ oligodendrocyte survival.

**Conclusion:**

IL-1Ra improved EEG activity and markedly attenuated systemic inflammation, microgliosis and oligodendrocyte loss following LPS exposure in near-term fetal sheep. Further studies examining the long-term effects on brain maturation are now needed.

**Supplementary Information:**

The online version contains supplementary material available at 10.1186/s12974-021-02238-4.

## Background

Perinatal infection/inflammation is commonly associated with high mortality and poor neurodevelopmental outcomes such as cerebral palsy that can have devastating lifelong impact [1–3]. Thus, there is a considerable need to develop interventions to prevent disability after infection/inflammation to reduce the growing economic and social burdens of perinatal brain injury on the affected individuals, their families and society [4, 5].

Experimentally, exposure to bacterial products such as lipopolysaccharide (LPS) is consistently associated with white matter injury [6–8]. In turn, in both term and preterm infants, diffuse/punctate white matter lesions are strongly associated with impaired postnatal brain growth [9] and impaired neurodevelopment [10]. Most studies of perinatal infection have focussed on the preterm brain, but infection is also common in near-term and term infants both in isolation and in combination with acute hypoxia-ischemia in low-, middle- [11] and high-income countries [12]. Moreover, preclinical studies indicate that therapeutic hypothermia may not be neuroprotective after exposure to gram-negative infection/inflammation [13–15]. Thus, more effective interventions that target specific injury pathways for neuroinflammation are needed to improve neurodevelopmental outcomes.

Although multiple pathways are involved in perinatal infection/inflammation, the pro-inflammatory cytokine interleukin-1β (IL-1β) is consistently upregulated in human and experimental perinatal encephalopathy [16–19]. Further, elevated cord blood levels of IL-1β are associated with impaired cerebral metabolism and developmental delay at 2 years of age [20] and increased systemic IL-1β levels are associated with white matter injury in neonatal piglets exposed to LPS [21, 22]. Collectively, these data support the hypothesis that IL-1β inhibition may be a viable therapeutic target for attenuating neural injury after perinatal infection/inflammation.

In this study, we tested the hypothesis that IL-1 inhibition started 1 h after LPS exposure with a clinically available IL-1 receptor antagonist (IL-1Ra), Anakinra, would reduce the severity of neuroinflammation and brain injury in near-term (0.85 gestation) fetal sheep. At this age, brain development in sheep is broadly equivalent to the near-term/term human infant [23]. A paradigm of progressively increasing doses of LPS was used in this study to reflect the typical progressive increase in inflammation during perinatal infection [24, 25].

## Materials and methods

All procedures were approved by the Hudson Institute of Medical Research Animal Ethics committee and were conducted in accordance with the National Health and Medical Research Council Code of Practice for the Care and Use of Animals for Scientific Purposes (Eighth Edition). Twenty-six pregnant Border-Leicester ewes bearing singleton or twin fetuses underwent aseptic surgery on either day 124 or 125 days gestation. Food but not water was withdrawn approximately 18 h before surgery. Anaesthesia was induced by i.v. injection of sodium thiopentone (20 mL) and maintained using 2-3% isoflurane in oxygen (Bomac Animal Health, New South Wales, Australia). Ewes received prophylactic antibiotics (ampicillin, 1 g i.v.; Austrapen, Lennon Healthcare, St. Leonards, New South Wales, Australia, and engemycin, 500 mg i.v.; Schering-Plough, Upper Hutt, New Zealand) immediately before surgery. Isoflurane levels, heart rate and respiratory rate were continuously monitored throughout surgery by trained anaesthetic staff.

### Fetal instrumentation

A midline maternal laparotomy was performed, the fetus was exposed and polyvinyl catheters were inserted into the right brachiocephalic artery, brachial vein and amniotic cavity. In the case of a twin pregnancy, only one twin was instrumented. An ultrasonic flow probe (3 mm; Transonic Systems, Ithaca, NY, US) was implanted around the carotid artery, enabling continuous monitoring of carotid artery blood flow (CaBF), as a surrogate for brain blood flow [26]. Two pairs of electroencephalograph (EEG) electrodes (AS633-7SSF; Cooner Wire, Chatsworth, CA, USA) were placed through burr holes onto the dura over the parasagittal parietal cortex (10 and 20 mm anterior to bregma, and 10 mm lateral) and secured using surgical bone wax and cyanoacrylate glue. A pair of electrodes was sewn into the nuchal muscle to record electromyographic (EMG) activity as a measure of fetal movement. The fetus was returned to the uterus in its original orientation and all fetal leads were exteriorised through the maternal flank. A catheter was inserted into the maternal jugular vein for administration of post-operative antibiotics and euthanasia at the end of the experimental period. At the completion of surgery, ewes received fentanyl for 3 days via a transdermal patch placed on the left hind leg (75 μg/h; Janssen Cilag, North Ryde, New South Wales, Australia).

Ewes were housed together in separate metabolic crates in a temperature-controlled room (20 ± 2 °C and relative humidity of 50 ± 10%) with a 12-h light-dark cycle with ad libitum access to food and water. Four to five days of postoperative recovery was allowed before experiments commenced. Ewes and fetuses received daily i.v. infusions of ampicillin (800 mg, maternal i.v. and 200 mg, fetal i.v.) and engemycin (500 mg, maternal i.v.) for three consecutive days after surgery. Fetal catheters were maintained patent with a continuous infusion of heparinised saline (25 IU/mL) at a rate of 0.2 mL/h.

### Experimental recordings

Continuous recordings of fetal mean arterial blood pressure (MAP), amniotic pressure, CaBF, fetal heart rate (FHR, derived from the beat-to-beat interval of the carotid artery pulse), EEG and nuchal EMG began 24 h prior to the first saline/LPS infusion (at 129 days of gestation) and continued until the end of the experiment (at 134 days of gestation). Amniotic and mean arterial pressures were measured using pressure transducers (ADInstruments, Castle Hill, New South Wales, Australia). The arterial blood pressure signal was collected at 1 kHz using a mains filter. The analogue fetal EEG signal was band pass filtered with cut-off frequencies set at 1 and 20 Hz and digitised at a sampling frequency of 400 Hz. EEG power was derived from the analogue signal, whilst the spectral edge was calculated as the frequency below which 90% of the intensity was present. For data presentation, total EEG power (dB) was normalised by log transformation (20 × log intensity) [27, 28]. The analogue fetal EMG signal was high pass filtered with a cut-off frequency of 100 Hz and digitised at a sampling frequency of 2 kHz.

### Experimental protocol

Experiments started at 129 days of gestation. Fetuses were randomly allocated to three groups: control (vehicle (saline), *n* = 9), LPS (*Escherichia coli*, 055:B5, MilliporeSigma, MO, USA) + vehicle (*n* = 8) and LPS + IL-1Ra (Anakinra, 13 mg/kg i.v. dissolved in saline, *n* = 9). The dose was guided by previous pharmacokinetic and neuroprotection studies in non-human primates and humans that administered Anakinra i.v. at a dose of 1.4-10 mg/kg [29, 30]. The final dose of IL-1Ra was decided following preliminary trials using our preclinical model of progressive LPS-induced inflammation where we compared i.v. doses of 4-13 mg/kg. In these preliminary trials, the 13 mg/kg dose was associated with the greatest reduction in periventricular microgliosis. Thus, 13 mg/kg was chosen for the present study.

Fetuses received 300 ng, 600 ng and 1200 ng infusions of LPS diluted in 2 mL of saline i.v. (infusion rate: 1 mL/min) at 0 h, 24 h and 48 h, respectively. This experimental model is relevant to the acute inflammatory exacerbations observed during perinatal infection/inflammation, which is associated with adverse neurodevelopmental outcomes [31, 32]. Controls received an equivalent volume of saline at the same infusion rate. Infusions of IL-1Ra (Anakinra, Sobi, Stockholm, Sweden) began 1 h after LPS administration on each consecutive day (i.e. 1, 25 and 49 h, respectively), at a rate of 0.75 mL/h over 4 h. Fetal preductal arterial blood samples were collected every morning (0900 h) starting from 30 min before the start of the experiment until the day of post-mortem for pH, blood gases and glucose and lactate concentrations (ABL 90 Flex Plus analyser, Radiometer, Brønshøj, Denmark).

**Fig. 1:**
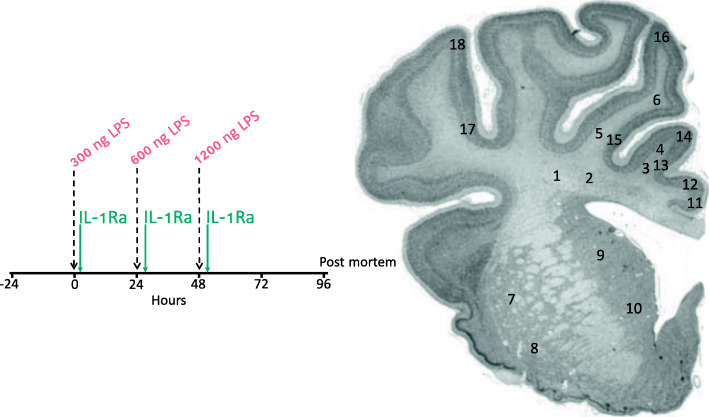
Schematic outlining the study design. The study consisted of 3 groups: control (vehicle, *n* = 9), LPS+vehicle (*n* = 8) and LPS+IL-1Ra (*n* = 9). The dashed vertical lines show the timing of LPS/vehicle infusions. The vertical green lines show the timing of IL-1Ra infusions (13 mg/kg) which were started 1 h after LPS infusions. Controls received an equivalent volume of vehicle (saline) during the infusion periods. Continuous physiological recordings were performed throughout the experimental period. Fetal preductal arterial blood was collected immediately before LPS or vehicle infusions and + 2 and + 6 h thereafter for measurement of cytokine levels and blood biochemistry. At 96 h, brains were collected for histological assessment. Numbers indicate regions of interest (ROIs) for assessment of the periventricular (1, 2), first (3, 4) and second (5, 6) intragyral white matter tracts, caudate nucleus (7, 8), putamen (9, 10), cingulate cortex (11, 12), first (13, 14) and second (15, 16) parasagittal cortices and the lateral cortex (17, 18)

Four days after the start of infusions, sheep were euthanized by intravenous injection of pentobarbitone sodium (Lethabarb, Virbac, New South Wales, Australia). The study protocol is illustrated in Fig. 1.

### Fetal cytokine measurements

Additional blood samples were collected immediately before LPS or saline infusions, and 2 and 6 h after LPS/saline infusions for measurement of cytokine levels using commercially available bovine assays that cross-react with sheep. Plasma levels of IL-1β, IL-6, IL-10 and tumour necrosis factor (TNF) were quantified using a Milliplex MAP bovine cytokine magnetic bead panel assay kits (cat#: BCYT1-33K; MerckMillipore, Burlington, MA, USA). Internal quality controls were included in each assay and cytokine levels were within the detection limit in all samples. Standards were bovine recombinant IL-1β (range, 2.6-40,000 pg/mL; assay sensitivity, 2.16 pg/mL), TNF (range, 12.8-200,000 pg/mL; assay sensitivity, 10.88 pg/mL), IL-6 (range, 2.6-40,000 pg/mL; assay sensitivity, 3.56 pg/mL) and IL-10 (range, 0.96-15,000 pg/mL; assay sensitivity, 0.57 pg/mL). Curve sensitivity values derived from the cross reactivity (bovine and sheep) analysis were as follows: IL-1β, 3.39 pg/mL; TNF, 19.07 pg/mL; IL-6, 1.14 pg/mL and IL-10, 0.73 pg/mL (data provided by MerckMillipore). Time points chosen for cytokine analysis were based on previous studies using similar experimental paradigms [7, 33]. In brief, 96 well plates were washed and then coated with the sample, assay buffer, serum matrix and antibody-immobilised beads. The plates were left to incubate overnight at 4 °C. The plates were washed and filled with the detection antibodies for 1 h. Streptavidin-phycoerythrin was added to the plates for 30 min. Finally, sheath fluid was added to the plates and cytokine concentrations were quantified using a Bio-Plex MAGPIX® Multiplex reader with xPOTENT® software (Bio-Rad, CA, USA).

Plasma levels of IL-1α were assessed using an ovine-specific IL-1⍺ enzyme-linked immunosorbent assay (ELISA) kit (cat#: ELO-IL1A; RayBiotech, Peachtree Corners, GA, USA) according to manufacturer’s instructions. In brief, samples were added to the 96 well plates for 2.5 h at room temperature. The plates were washed and incubated with the biotinylated antibody for 1 h at room temperature. Streptavidin was added to the plates for 45 min at room temperature, followed by TMB one-step substrate for 30 min at room temperature. Finally, the reaction was stopped in 0.2 M sulphuric acid and quantified at 450 nm on a plate reader (SpectraMax i3, Molecular Devices, San Jose, CA, USA).

Cerebral spinal fluid (CSF) levels of IL-1Ra were quantified using a human-specific IL-1Ra ELISA (Abcam, Cambridge, UK, Cat# Ab211650). In brief, samples and antibodies were added to the 96 well plates and incubated for 1 h at room temperature. The plate was washed and TMB was added to the wells for 10 min in the dark at room temperature. Finally, the reaction was stopped in the stop solution and quantified at 450 nm on a plate reader (SpectraMax i3, Molecular Devices).

### CSF and brain collection and processing

At post-mortem, a subset of subjects (*n* = 3/group) underwent cisternae magna puncture for cerebrospinal fluid collection. The right hemisphere underwent immersion fixation with 10% phosphate-buffered formalin for 7 days before processing and embedding using a standard paraffin tissue preparation. Using a brain mould, the right hemisphere was cut with a blocking blade into 5-mm thick coronal blocks. Blocks from the forebrain, approximately 23 mm anterior to stereotaxic zero, with a clearly visible cortex, striatum and intragyral and periventricular white matter tracts were sectioned using a microtome (Leica Microsystems, Victoria, Australia) into 8-μm thick coronal sections. Periventricular white matter from the left hemisphere was dissected and snap-frozen in liquid nitrogen and stored at −80 °C.

### Immunohistochemistry

Slides were baked at 60 °C for 1 h then dewaxed in xylene, rehydrated in increasing concentrations of ethanol and washed in 0.1 mol/L phosphate-buffered saline (PBS). Antigen retrieval was performed in citrate buffer (pH 6) using a microwave for 15 min. Endogenous peroxide quenching was performed by incubating slides in 0.1% H_2_O_2_ in methanol. Non-specific antigen blocking was performed using 3% normal goat serum. Sections were labelled with 1:200 rabbit anti-glial fibrillary acidic protein (GFAP; Abcam, cat#: ab68428), 1:200 rabbit anti-ionised calcium binding adaptor molecule 1 (Iba-1, Abcam, cat#: ab153696), 1:200 rabbit anti-oligodendrocyte transcription factor 2 (Olig-2, for oligodendrocytes at all stages of development; Abcam, cat#: ab42453;), 1:200 mouse anti-cyclic nucleotide 3′ phosphodiesterase (CNPase, for immature and mature oligodendrocytes; Abcam, cat#: ab6319), 1:200 rat anti-myelin basic protein (MBP, for myelin density; MerkMillipore, cat#: MAB395), 1:200 mouse anti-adenomatous polyposis coli clone (CC1, for mature oligodendrocytes, MerckMillipore, cat#: OP80-100UG), 1:800 rabbit anti-cleaved caspase3 (Abcam, cat#: ab2302), 1:200 rabbit anti-neuronal nuclei (NeuN, Abcam, cat#: ab177487) and 1:250 rabbit anti-IL-1β (cat#: NB600-633, Novus, CO, USA) overnight at 4 °C. Sections were incubated in biotin conjugated IgG (1:200, goat anti-rabbit (Dako, Victoria, Australia), goat anti-mouse or goat anti-rat secondary antibodies (Vector Laboratories, CA, USA) for 3 h at room temperature before being incubated in avidin-biotin complex (Sigma-Aldrich) for 45 min at room temperature. Sections were reacted with 3,3′-diaminobenzidine tetrahydrochloride (Sigma-Aldrich). The reaction was stopped in PBS before slides were dehydrated in xylene and increasing concentrations of ethanol, mounted in dibutylphthalate polystyrene xylene and cover slipped.

Astrocytes (GFAP+ cells), microglia (Iba-1+ cells), oligodendrocytes (Olig-2+, CNPase+ and CC1+ cells), myelin density (MBP and CNPase area fraction), apoptosis (caspase 3+ cells) and neurons (NeuN+ cells) were visualised using light microscopy (Olympus, Tokyo, Japan) at 40× magnification and cellSens imaging software (Version 2.3, Olympus). Positive cells or immunoreactivity were quantified for each region of interest from 2 sections per subject using the ImageJ software (v2.00, LOCI, University of Wisconsin). The IL-1β immunoreactivity scoring system was adapted from Girard S et al. [16]. Scoring was based on the intensity of staining (1 = light, 2 = moderate, 3 = moderate-to-intense and 4 = intense). Microglia (Iba-1+ cells) showing ramified (small cell body with > 1 branching process) or amoeboid morphology (large cell bodies, with ≤ 1 branching process) were included in our assessment [7, 34]. Caspase-3+ cells displaying both immunostaining and apoptotic bodies were counted. For each region of interest, average scores from two slides from the right hemisphere were calculated. All imaging and cell counts were performed by an assessor who was blinded to the treatment group by coding.

Immunofluorescence was used to double label total Olig-2 and Caspase 3. Antigen retrieval was performed using a Dako PT Link Antigen Retrieval System using S1699 target retrieval solution (Dako, Santa Clara, CA, USA) for 30 min at 98 °C. Sections were then washed in buffer solution (K8000, Dako) for 5 min at room temperature. Staining was performed using a Dako AutostainerPlus. Non-specific antigens were blocked using a protein block serum (X0909, Dako) for 1 h at room temperature. Sections were labelled with 1:100 mouse anti-Olig-2 (MerckMillipore; Cat#: MABN50) and 1:100 rabbit anti-cleaved caspase-3 (Abcam, cat#: ab2302) for 60 min at room temperature. Negative controls that did not contain the target antibody were included to confirm the absence of non-specific staining (Supplementary figure 1). Sections were incubated in 1:500 donkey anti-mouse-Alexa Fluor 488 (Cat#: 715-545-151, Jackson ImmunoResearch, West Grove, PA, USA) and 1:500 donkey anti-mouse-Alexa Fluor 647 (cat#: 711-605-152, Jackson ImmunoResearch) for 1 h at room temperature. Autofluorescence was quenched by incubating slides with 0.3% Sudan black in 70% ethanol for 30 s. Slides were washed with distilled water and coverslipped using ProLong Gold Antifade Mountant (cat# P36934, ThermoFisher, Waltham, MA, USA). Sections were imaged at 20× magnification using the QuPath imaging software (Version 0.2.3, [35]).

### Gene analysis

The periventricular white matter from the left hemisphere was homogenised and total mRNA was isolated using an RNeasy Midi Kit (QIAGEN, Venlo, Netherlands) and reverse transcribed into single stranded cDNA (SuperScript III First-Strand Synthesis System, Invitrogen, MA, USA). Genes of interest were measured by qRT-PCR using an Applied Biosystems Quantstudio 6 Real-Time PCR system. Relative mRNA expression of IL-1 α and IL-1β were measured. The expression of the genes of interest were normalised to the 18S RNA for each sample by subtracting the Ct value for 18S from the Ct value for the gene of interest (ΔCt). mRNA levels of genes of interest were normalised using the formula 2^−ΔCt^ and the results expressed as a fold change from control. A threshold value (Ct) for each sample was measured in triplicate and a control sample containing no cDNA template was included in each run. Details of primers are presented in Table 1.

**Table 1:** Primer sequences for qPCR

Gene	Species	Accession number	Primer sequence	Amplicon length, base pairs
*18S*	Rat	NR_046237.1	5′-GTAACCCGTTGAACCCCATT-3′ 3′-CCATCCAATCGGTAGTAGCG-5′	151
*IL-1α*	Sheep	NM_001009808.1	5′-GTCCATACATGACGGCTGCTA-3′ 3′-GGTGTCTCAGGCATCTCCTTAT-5′	184
*IL-1 β*	Sheep	NM_001009465.2	5′-CGATGAGCTTCTGTGTGATG-3′ 3′–CTGTGAGAGGAGGTGGAGAG-5′	121

### Data analysis and statistics

Offline analysis of physiological data was performed using the LabChart Pro software (v8.1.3; ADInstruments). Physiological data were processed as hourly averages for analysis and presentation. Physiological data are presented from 24 h before the first saline/LPS infusion until the end of the experiment. EEG power and frequency were normalised by subtracting the baseline average (24 h before the first saline/LPS infusion) from the absolute value. Due to a small but significant difference in baseline FHR between the groups, the relative change in FHR was calculated as the percentage change from the 24 h baseline period.

Fetal body and brain weights and periventricular white matter mRNA levels were analysed by one-way ANOVA. Blood biochemistry and physiological data were analysed using a two-way ANOVA. Physiological data from the baseline, LPS/saline infusion and recovery periods were analysed as separate time periods. Histological data were analysed by two-way ANOVA with treatment as an independent factor and brain region treated as a repeated measure. For physiological and neuropathological data, when statistical significance was found between groups or between groups and time/brain region, post hoc comparisons were made using the Fisher’s protected least significant difference test [36]. A power analysis for oligodendrocyte loss suggested the study had 90% power to detect a minimum difference of 20 cells/field, with an alpha of 0.05. Statistical significance was accepted when *P* < 0.05. Data are presented as means ± standard error (SE).

## Results

### Baseline period

**Fig. 2:**
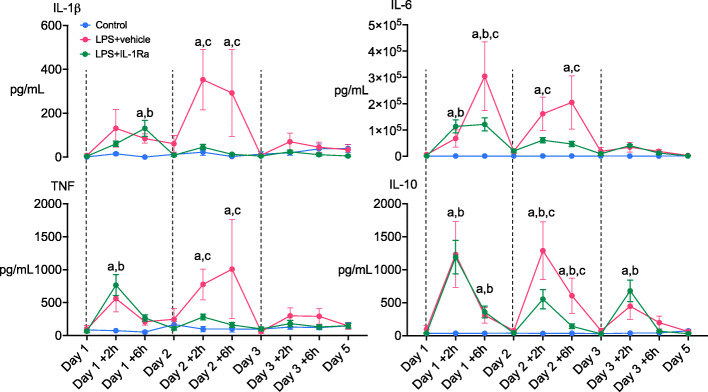
Time course of changes in plasma interleukins (ILs)-1β, 6, 10 and tumour necrosis factor (TNF) in control (blue circles, *n* = 9), LPS+vehicle (red circles, *n* = 8) and LPS+IL-1Ra (green circles, *n* = 9) groups. Dashed vertical black lines represent administration of LPS/vehicle infusions. Data are hourly means ± SE. *a* = *P* < 0.05 LPS+vehicle vs. control, *b* = *P* < 0.05 LPS+IL-1Ra vs. control, *c* = *P* < 0.05 LPS+vehicle vs. LPS + IL-1Ra

**Fig. 3:**
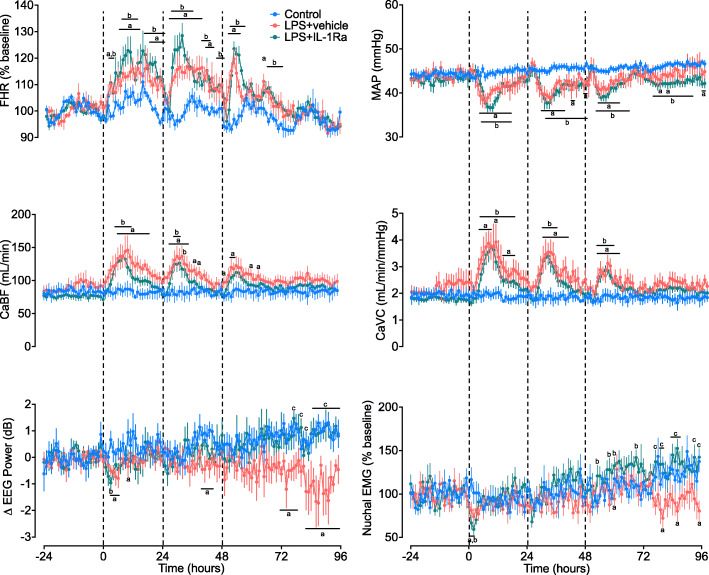
Cardiovascular, cerebrovascular and neurophysiological changes over time. Mean arterial pressure (MAP), fetal heart rate (FHR), carotid artery blood flow (CaBF) and vascular conductance (CaVC), EEG power and nuchal electromyography (EMG) in control (blue circles, *n* = 9), LPS+vehicle (red circles, *n* = 8), and LPS+IL-1Ra (green circles, *n* = 9) groups. Data are hourly means ± SE. *a* = *P* < 0.05 control vs. LPS+vehicle, *b* = *P* < 0.05 LPS+ IL-1Ra vs. control, *c* = *P* < 0.05 LPS+vehicle vs. LPS+IL-1Ra < 0.05 LPS+vehicle vs. LPS + IL-1Ra

Prior to LPS exposure, circulating levels of cytokines, cardiovascular and neurophysiological data and blood biochemistry did not differ between groups and were within the normal range for our laboratory (Figs. 2 and 3, and Table 2).

**Table 2:** Arterial pH, blood gases, glucose and lactate concentrations. Data are mean ± SE

	D1 base	D1+2 h	D1+6 h	D2 base	D2+2 h	D2+6 h	D3 base	D3+2 h	D3+6 h	D4 base	D5 base
pH											
Saline	7.38 ± 0.01	7.38 ± 0.01	7.38 ± 0.01	7.37 ± 0.01	7.38 ± 0.01	7.38 ± 0.01	7.38 ± 0.01	7.38 ± 0.01	7.37 ± 0.01	7.37 ± 0.01	7.36 ± 0.01
LPS	7.36 ± 0.01	7.32 ± 0.01*	7.34 ± 0.01*	7.35 ± 0.01*#	7.33 ± 0.01*	7.35 ± 0.01*	7.34 ± 0.01*	7.33 ± 0.01*	7.34 ± 0.01*	7.32 ± 0.01*	7.34 ± 0.01
LPS+IL-1Ra	7.38 ± 0.01	7.33 ± 0.01*	7.36 ± 0.01	7.38 ± 0.01	7.34 ± 0.01*	7.37 ± 0.01	7.36 ± 0.01	7.34 ± 0.01*	7.35 ± 0.01	7.35 ± 0.01	7.36 ± 0.01
PaCO_2_											
Saline	46.6 ± 1.0	45.4 ± 1.0	46.2 ± 1.0	47.2 ± 1.0	46.9 ± 1.4	46.0 ± 1.1	47.4 ± 0.6	46.7 ± 0.8	46.9 ± 1.2	48.0 ± 1.0	47.7 ± 1.1
LPS	49.1 ± 0.4	49.7 ± 1.0*	51.5 ± 1.2*	49.4 ± 0.8	51.7 ± 0.9*	50.7 ± 0.9*	47.7 ± 0.9	48.5 ± 1.2	48.5 ± 0.6	46.0 ± 1.3	48.4 ± 1.0
LPS+ IL-1Ra	47.0 ± 1.2	48.5 ± 1.4	49.6 ± 1.0*	49.3 ± 0.8	50.2 ± 1.0*	50.8 ± 1.1*	48.8 ± 0.8	48.3 ± 1.0	48.4 ± 0.7	48.1 ± 1.2	49.2 ± 0.7
PaO_2_											
Saline	23.9 ± 1.2	23.8 ± 0.9	23.3 ± 1.5	24.2 ± 1.1	23.6 ± 0.8	25.2 ± 0.9	22.5 ± 1.5	24.3 ± 1.1	24.0 ± 1.4	24.2 ± 1.3	23.9 ± 0.8
LPS	22.1 ± 1.0	22.8 ± 0.8	19.2 ± 0.6*	22.9 ± 1.2	21.1 ± 1.2	20.1 ± 1.3*	22.8 ± 1.1	21.6 ± 1.3	20.8 ± 0.6*	23.5 ± 1.4	23.1 ± 0.7
LPS+ IL-1Ra	21.3 ± 0.5	20.0 ± 0.6*	17.8 ± 0.3*	19.3 ± 0.9*	17.8 ± 1.2*	16.7 ± 1.0*	20.8 ± 0.9	19.0 ± 1.0	18.3 ± 0.7*	20.7 ± 1.0	21.2 ± 1.0
sO_2_											
Saline	66 ± 2	68 ± 2	65 ± 3	65 ± 2	66 ± 2	69 ± 2	64 ± 3	68 ± 2	66 ± 2	65 ± 2	65 ± 2
LPS	62 ± 3	59 ± 3*	47 ± 2*	59 ± 3	50 ± 4*	48 ± 4*	61 ± 3	56 ± 3*	57 ± 2*	65 ± 2	65 ± 3
LPS+ IL-1Ra	64 ± 1	57 ± 3*	51 ± 2*	56 ± 3	50 ± 4*	48 ± 3*	62 ± 2	55 ± 3*	53 ± 2*	63 ± 3	61 ± 2
Glucose											
Saline	0.9 ± 0.0	0.9 ± 0.0	0.9 ± 0.0	0.9 ± 0.0	0.9 ± 0.0	0.9 ± 0.0	0.9 ± 0.1	0.9 ± 0.1	0.9 ± 0.1	0.9 ± 0.0	0.9 ± 0.0
LPS	0.9 ± 0.1	1.1 ± 0.1	0.8 ± 0.1	0.9 ± 0.0	0.8 ± 0.0	0.9 ± 0.2	1.0 ± 0.1	0.9 ± 0.1	1.0 ± 0.0	1.1 ± 0.2	0.9 ± 0.1
LPS+ IL-1Ra	0.9 ± 0.1	0.9 ± 0.1	0.8 ± 0.1	0.9 ± 0.1	0.8 ± 0.1	0.9 ± 0.1	1.0 ± 0.1	0.8 ± 0.1	1.0 ± 0.1	0.9 ± 0.1	0.9 ± 0.1
Lactate											
Saline	1.7 ± 0.2	1.5 ± 0.1	1.5 ± 0.1	1.5 ± 0.1	1.5 ± 0.1	1.5 ± 0.1	1.5 ± 0.1	1.5 ± 0.2	1.5 ± 0.1	1.4 ± 0.1	1.5 ± 0.1
LPS	1.7 ± 0.1	3.1 ± 0.3*	4.0 ± 0.4*	1.5 ± 0.1	2.3 ±0.1*	1.9 ± 0.2	1.2 ± 0.1	1.3 ± 0.1	1.3 ± 0.1	1.1 ± 0.1	1.1 ± 0.1
LPS+ IL-1Ra	1.6 ± 0.1	2.9 ± 0.4*	4.4 ± 0.9*	1.5 ± 0.2	2.1 ± 0.4	2.1 ± 0.4	1.2 ± 0.1	1.5 ± 0.1	1.3 ± 0.1	1.1 ± 0.1	1.2 ± 0.1

*P < 0.05 vs. control

### Fetal biochemistry

In the LPS+vehicle group, pH was lower than in controls from day 1 to 4 (*P* < 0.05; day 1 +2 h to day 4, Table 2). In LPS+IL-1Ra-treated fetuses, pH was lower than control at +2 h on days 1, 2 and 3 after LPS infusions. PaCO_2_ was higher in LPS+vehicle and LPS+IL-1Ra-treated fetuses between days 1 and 2 after LPS infusion (*P* < 0.05 vs. control, day 1 +2 h to day 2 +6 h). PaO_2_ and sO2 were lower in LPS+vehicle and LPS+IL-1Ra groups between days 1 and 3 after LPS infusions (*P* < 0.05 vs. control; day 1 +2h to day 3). Arterial lactate was higher in LPS+vehicle and LPS+IL-1Ra-treated groups after the first LPS infusion (day 1 +2 h and +6 h, *P* < 0.05 vs. control). After the second LPS infusion, arterial lactate was higher in the LPS+vehicle-treated group compared to controls (*P* < 0.05; day 2 +2 h, Table 2). There were no differences in pH, blood gases, glucose and lactate concentrations between LPS+vehicle and LPS+IL-1Ra-treated groups throughout the study period (Table 2).

### Plasma and cerebrospinal fluid cytokines

Plasma IL-1β levels increased 6 h after the first LPS infusion in the LPS+vehicle and LPS + IL-1Ra groups compared to controls (*P* < 0.05, Fig. 2). After the second LPS infusion, IL-1β was higher in the LPS+vehicle group compared to control and LPS+IL-1Ra groups at +2 h and +6 h (*P* < 0.05, day 2 +2 and +6 h, Fig. 2). IL-1⍺ was not detectable in plasma samples from any of the groups (data not shown). Plasma TNF levels greater than control levels at +2 h after the first and second LPS infusions in the LPS+vehicle and LPS+IL-1Ra groups (*P* < 0.05, day 1 +2 h and day 2 +2 h). At +6 h after the second LPS infusion, TNF was higher in the LPS+vehicle group compared to LPS+IL-1Ra and control groups (*P* < 0.05, day 2 +6 h, Fig. 2). Plasma IL-6 was higher in LPS+vehicle and LPS+IL-1Ra groups compared to control at +2 h and +6 h after the first LPS infusion. After the second LPS infusion, IL-6 levels were higher in the LPS+vehicle group compared to LPS+IL-1Ra and control groups (*P* < 0.05, Fig. 2). Plasma IL-10 levels were increased in the LPS+vehicle and LPS+IL-1Ra groups compared to controls at +2 and +6 h after the first LPS infusion (*P* < 0.05 vs. control, day 1 +2 and +6 h, Fig. 2). After the second LPS infusion, IL-10 was higher in the LPS+vehicle group compared to the LPS+IL-1Ra and control groups at +2 h and +6 h (*P* < 0.05, day 2 +2 h and +6 h, Fig. 2). After the third LPS infusion, IL-10 was higher in the LPS+vehicle and LPS+IL-1Ra groups at +2 h compared to control (*P* < 0.05, day 3 +2 h, Fig. 2). In a subset of subjects in whom CSF samples were collected at post-mortem (*n* = 3/group), IL-1Ra levels [mean (range)] were not detectible in controls, 1568 (1092-2044) pg/mL in the LPS+vehicle group and 639 (243-1088) pg/mL in the LPS+IL-1Ra group.

### Fetal physiological changes

#### Fetal heart rate

After the first LPS infusion, FHR was increased in the LPS+vehicle and LPS+IL-1Ra groups compared to controls from 2 to 23 h (*P* < 0.05, Fig. 3). After the second LPS infusion, FHR was increased in the LPS+vehicle group compared to controls from 26 to 44 h (i.e. 2-20 h after the second LPS infusion, *P* < 0.05). In the LPS+IL-1Ra group, FHR was increased compared to control between 27 and 47 h (i.e. 3-23 h after the second LPS infusion, *P* < 0.05). After the third LPS infusion, FHR was significantly higher in the LPS + vehicle group between 50 and 54 h, and at 64 h compared to control (i.e. 2-6 h and 16 h after the third LPS infusion, *P* < 0.05). In the LPS + IL-1Ra group, FHR was higher between 51 and 56 h, and at 65-71 h, compared to control (3-8 h and 17-23 h after the third LPS infusion, *P* < 0.05). During recovery, FHR did not differ between groups (Fig. 3).

#### Mean arterial blood pressure

After the first LPS infusion, mean arterial blood pressure (MAP) was reduced in the LPS+vehicle group between 4 and 16 h compared to control (*P* < 0.05, Fig. 3). In the LPS+IL-1Ra group, MAP was reduced compared to controls from 5 to 16 h (*P* < 0.05). After the second LPS infusion, MAP was reduced in the LPS+vehicle group from 29 to 38 h, 41-42 h and at 46-47 h (i.e. 5-14 h, 17-18 h and 22-23 h after the second LPS infusion, respectively). In the LPS+IL-1Ra-treated group, MAP was reduced compared to controls from 31 to 47 h (i.e. 7-23 h after the second LPS infusion, *P* < 0.05). After the third LPS infusion, MAP was reduced in the LPS+vehicle group compared to control from 52 to 60 h (i.e. 4-12 h after the third LPS infusion, *P* < 0.05, Fig. 3). In the LPS+IL-1Ra group, MAP was lower than controls from 51 and 64 h (i.e. 3-16 h after the third LPS infusion, *P* < 0.05). During the recovery period, MAP was reduced in the LPS+vehicle group compared to control at 74, 80 and 95-96 h (*P* < 0.05). In the LPS+IL-1Ra group MAP was reduced compared to controls from 74 to 90 h (*P* < 0.05, Fig. 3).

#### Carotid arterial blood flow and vascular conductance

After the first LPS infusion, carotid arterial blood flow (CaBF) was higher in the LPS+vehicle group compared to control from 6 to 18 h (*P* < 0.05, Fig. 3). In the LPS + IL-1Ra-treated group, CaBF was higher than controls from 5 to 11 h (*P* < 0.05). After the second LPS infusion, CaBF was higher in the LPS + vehicle group compared to controls from 27 to 34 h, and at 37 and 39 h (i.e. 3-10 h, and 13 and 15 h after the second LPS infusion, *P* < 0.05). In the LPS + IL-1Ra group, CaBF was higher than controls from 29 to 31 h and at 33 h (i.e. 5-7 h and 9 h after the second LPS infusion, *P* < 0.05). After the third LPS infusion, CaBF was higher in the LPS+vehicle group compared to controls at 49 h, 52-53 h, 60 and 63 h (i.e. 1 h, 4-5 h, 12 and 15 h after the third LPS infusion, *P* < 0.05). There were no differences in CaBF between groups throughout the recovery period.

After the first LPS infusion, carotid arterial vascular conductance (CaVC) was higher than controls from 3 to 8 h, at 10 h and 13-17 h (*P* < 0.05). In the LPS+IL-1Ra group, CaVC was higher than controls from 4 to 18 h (*P* < 0.05). After the second LPS infusion, CaVC was higher in the LPS+vehicle group compared to controls from 29 to 40 h (i.e. 5-16 h after the second LPS infusion, *P* < 0.05). In the LPS + IL-1Ra group, CaVC was higher than control between 29 and 35 h (i.e. 5-11 h after the second LPS infusion, *P* < 0.05). After the third LPS infusion, CaVC was higher in the LPS+vehicle group compared to controls from 52 to 60 h (i.e. 4-12 h after the third LPS infusion, *P* < 0.05). In the LPS + IL-1Ra-treated group, CaVC was higher than controls from 51 to 56 h (i.e. 3-8 h after the third LPS infusion, *P* < 0.05, Fig. 3).

#### EEG power and frequency

After the first LPS infusion, EEG power fell in the LPS+vehicle group compared to controls from 3 to 6 h and at 10 h (*P* < 0.05, Fig. 3). In the LPS+IL-1Ra group, EEG power was lower than control at 3 h (*P* < 0.05). After the second LPS infusion, EEG power was lower in the LPS+vehicle group compared to controls from 40 to 44 h (i.e. 16-20 h after the second LPS infusion, *P* < 0.05). During the recovery period, EEG power was lower in the LPS + vehicle group compared to control at 72-78 h and 82-96 h (*P* < 0.05). In the LPS + IL-1Ra group, EEG power was higher compared to LPS+vehicle at 77-82 h, and 85-96 h (*P* < 0.05 vs. control, Fig. 3). There were no significant differences in EEG frequency between groups throughout the study period (data not shown).

#### Nuchal EMG

After the first LPS infusion, nuchal EMG activity was reduced in the LPS+vehicle and LPS+IL-1Ra groups compared to controls between 1 and 2 h (*P* < 0.05, Fig. 3). During the recovery period, nuchal EMG activity was lower in the LPS+vehicle group compared to controls at 60, 80, 86 and 96 h (*P* < 0.05, Fig. 3). In the LPS+IL-1Ra group, nuchal EMG activity was higher than control at 53, 58, 60 and 69 h (*P* < 0.05, Fig. 3). In the LPS+IL-1Ra group, nuchal EMG activity was higher compared to LPS+vehicle at 77, 79-80, 84-87, 94 and 96 h (*P* < 0.05, Fig. 3).

#### Post-mortem findings

There were no significant differences in body weight, brain weight or the ratio of males to females between the groups (Table 3).

**Table 3:** Fetal body weights, brain weights and sex

	Body weight (kg)	Brain weight (g)	Sex (M:F)
Control	4.5 ± 0.2	51.5 ± 1.0	6:3
LPS + vehicle	4.6 ± 0.2	48.8 ± 1.2	7:1
LPS + IL-1Ra	4.7 ± 0.3	49.6 ± 1.6	5:4

Data are means ± SE

### Gene analysis

**Fig. 4:**
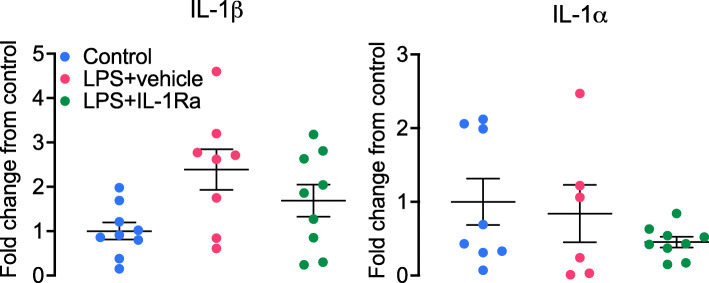
Interleukin (IL)-1β and IL-1⍺ mRNA levels in the periventricular white matter in controls (blue, *n* = 9), LPS + vehicle (red, IL-1β *n* = 8, IL-1⍺ *n* = 6, 2 subjects had undetectable values) and LPS + IL-1Ra (green, *n* = 9). Data are means ± SE and are expressed as the fold change from the mean control values

In the periventricular white matter, mRNA expression of IL-1β at 96 h was increased in the LPS+vehicle group compared to controls (*P* < 0.05, Fig. 4). IL-1β mRNA expression was not significantly lower in the LPS+IL-1Ra group compared to LPS+vehicle (*P* = 0.1). There were no differences in mRNA expression of IL-1⍺ between groups in the periventricular white matter (Fig. 4).

#### Histopathology

**Fig. 5:**
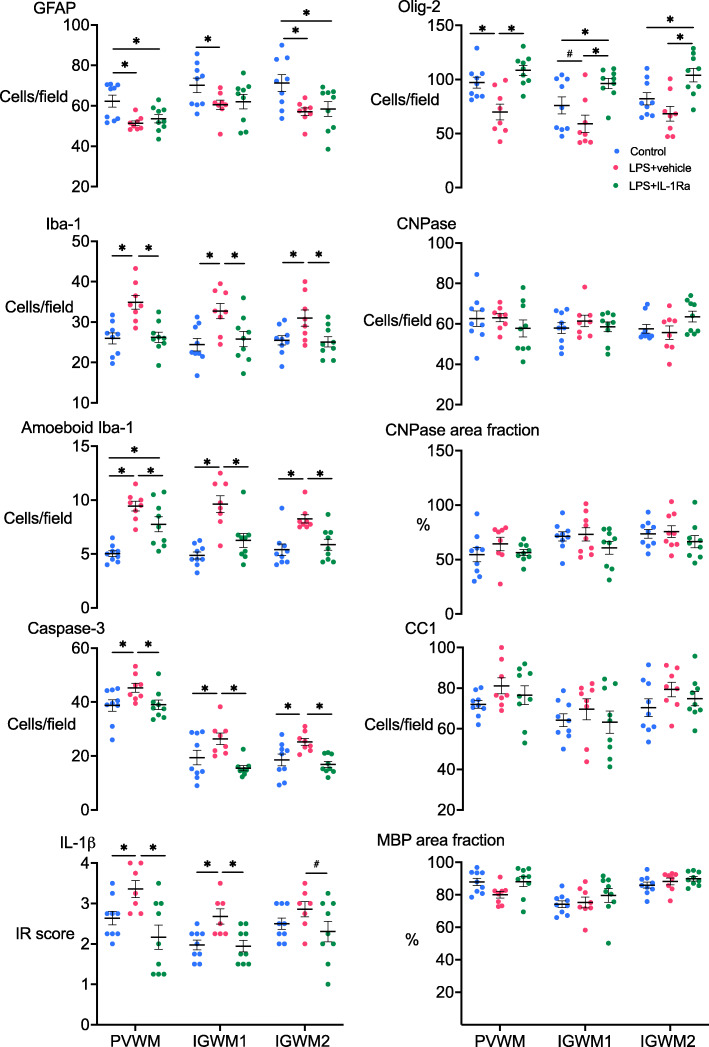
White matter immunohistochemistry. Glial fibrillary acidic protein (GFAP+), ionised calcium binding adaptor molecule (Iba-1+), amoeboid (Iba-1+) microglia, IL-1β immunoreactivity score, caspase 3, oligodendrocyte transcriptase factor-2 (Olig-2+) and 2′,3′-cyclic nucleotide 3′-phosphodiesterase (CNPase+) cell counts, % area fraction of CNPase staining, anti-adenomatous polyposis coli clone (CC1+) cell counts and % area fraction of myelin basic protein (MBP) in the periventricular white matter (PVWM), first and second intragyral white matter tracts (IGWM 1 and IGWM 2) in control (blue circles, *n* = 9), LPS+vehicle (red circles, *n* = 8) and LPS+IL-1Ra (green circles, *n* = 9) groups. Data are means ± SE. *P < 0.05 vs. control, #*P* = 0.06 vs. control

**Fig. 6:**
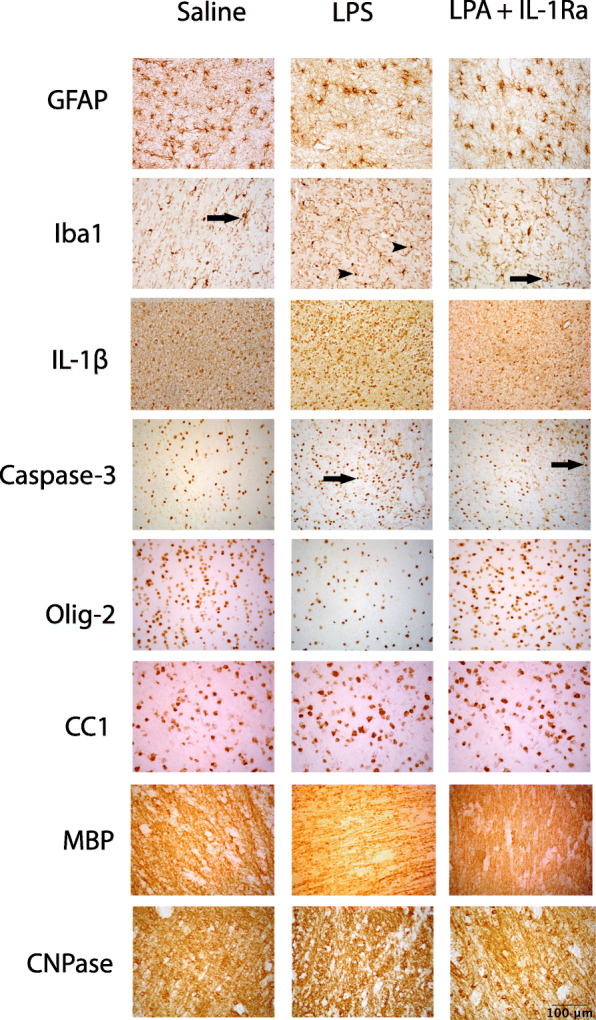
Representative photomicrographs showing positive staining of GFAP, Iba-1, IL-1β, caspase 3, Olig-2, CC1, MBP and CNPase in the periventricular white matter tracts. Scale bar = 100 μm. Arrows in the Iba-1 photomicrographs indicate microglia displaying a resting ramified phenotype, characterised by a small cell body with > 1 branching process. Arrowheads indicate microglia displaying an amoeboid morphology, characterised by a large cell body with ≤ 1 branching process. Arrows in the caspase-3 photomicrographs indicate positive cells displaying both staining and apoptotic bodies

The number of GFAP+ astrocytes was reduced in the periventricular and second intragyral white matter tracts in the LPS+vehicle and LPS+IL-1Ra groups compared to controls (*P* < 0.05, Figs. 5 and 6). In the first intragyral white matter tract, the number of GFAP+ astrocytes was reduced in the LPS+vehicle group compared to control. The numbers of Iba-1+ microglia and amoeboid microglia were increased in the periventricular and intragyral white matter tracts in the LPS+vehicle group compared to controls (*P* < 0.05). In the LPS+IL-1Ra group, the numbers of Iba-1+ microglia and amoeboid microglia were reduced in the white matter tracts compared to the LPS+vehicle group (*P* < 0.05, Figs. 5 and 6). IL-1β immunoreactivity was increased in the periventricular and first intragyral white matter tracts in the LPS+vehicle group compared to controls. In the LPS+IL-1Ra group, IL-1β immunoreactivity was reduced in the periventricular and first intragyral white matter tracts compared to the LPS+vehicle group (*P* < 0.05, Figs. 5 and 6).

The number of caspase 3+ cells was increased in the periventricular and intragyral white matter tracts in the LPS+vehicle group compared to control (*P* < 0.05). In the LPS+IL-1Ra group, the number of caspase 3 positive cells was reduced in the periventricular and intragyral white matter compared to the LPS+vehicle group (*P* < 0.05, Figs. 5 and 6).

The number of Olig-2+ oligodendrocytes was reduced in the periventricular white matter in the LPS + vehicle group compared to control (*P* < 0.05). In the first intragyral white matter tract, the number of Olig-2+ oligodendrocytes was not significantly reduced in the LPS+vehicle group compared to control (*P* = 0.06, Fig. 5). In the LPS + IL-1Ra group, the number of Olig-2+ oligodendrocytes was higher in the periventricular and intragyral white matter tracts compared to LPS+vehicle (*P* < 0.05, Figs. 5 and 6). In the intragyral white matter tracts, the number of Olig-2+ oligodendrocytes was higher in the LPS+IL-1Ra group compared to control (*P* < 0.05). The numbers of CNPase+ oligodendrocytes and area fraction of CNPase staining in the periventricular and intragyral white matter tracts did not differ between groups. Numbers of CC1+ oligodendrocytes and the area fraction of MBP staining in the periventricular and intragyral white matter were not significantly different between groups (Figs. 5 and 6).

**Fig. 7:**
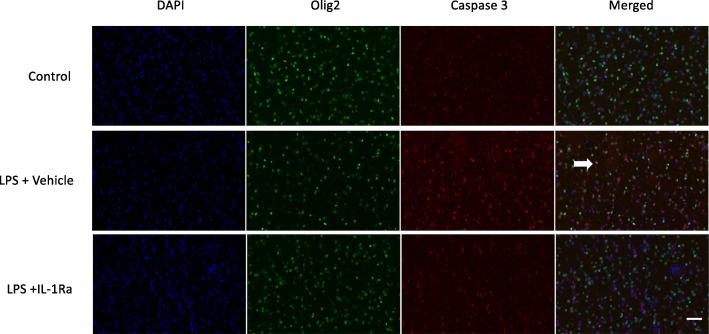
Representative photomicrographs showing immunofluorescent staining of 4′,6-diamidino-2-phenylindole (DAPI, showing cell nuclei, blue), oligodendrocyte transcriptase factor-2 (olig-2, green) and cleaved caspase-3 (red), and the merged image in the periventricular white matter of control, LPS+vehicle and LPS+IL-1Ra groups. Scale bar = 50 μm. Arrow indicates olig-2/caspase-3+ cell. Caspase-3+ oligodendrocytes appeared more abundant in the LPS+vehicle group compared to control and LPS+IL-1Ra-treated groups. The density of caspase-3/olig-2+ oligodendrocytes in the periventricular white matter of LPS+IL-1Ra-treated fetuses was similar to controls

In the periventricular white matter, caspase-3+ oligodendrocytes were more abundant in the LPS+vehicle group compared to the control and LPS+IL-1Ra-treated groups (Fig. 7). The density of caspase 3/olig-2+ oligodendrocytes in the periventricular white matter of LPS+IL-1Ra-treated fetuses appeared to be similar to controls (Fig. 7).

**Fig. 8:**
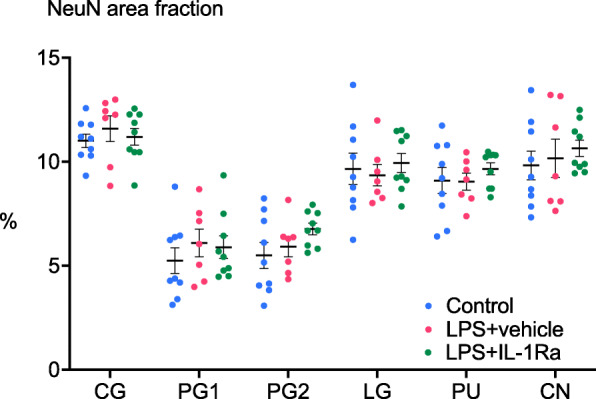
Neuronal survival in cortical and subcortical brain regions. Anti-neuronal nuclei (NeuN) % area staining in the cingulate gyrus (CG), first and second parasagittal gyri (PG 1 and PG 2), lateral gyrus (LG), caudate nucleus (CN) and putamen (PU) in control (blue circles, *n* = 9), LPS+vehicle (red circles, *n* = 8) and LPS+IL-1Ra (green circles, *n* = 9) groups. Data are means ± SE

There were no differences in the area fraction of NeuN staining between groups in the cingulate, parasagittal and lateral cortices, caudate nucleus and putamen (Fig. 8).

## Discussion

This present study demonstrates that IL-1β inhibition during progressive systemic LPS-induced inflammation in near-term fetal sheep reduced microgliosis and apoptosis, and improved survival of oligodendrocytes in the large white matter tracts. The reduction in neuroinflammation was associated with reduced circulating pro- and anti-inflammatory cytokines and improved recovery of EEG power and fetal movement after LPS-exposure.

Clinically, perinatal infection/inflammation is associated with a high risk of neonatal mortality and morbidity. In cases of perinatal infection/inflammation, upregulation of circulating IL-1β is associated with an increased risk of short- and long-term neurodevelopmental impairment after birth [37, 38]. Increased IL-1β expression has been detected in the cerebrospinal fluid of term neonates with encephalopathy and was strongly associated with impaired neurodevelopmental outcomes [39]. Furthermore, at post-mortem, neonates with white matter injury showed increased IL-1β expression localised to areas of white matter gliosis [17]. Similarly, increased circulating levels of IL-1β are associated with acute white matter injury and impaired neural metabolism [21, 22]. These data demonstrate a strong association between elevated systemic and central IL-1β production and perinatal brain injury. Furthermore, IL-1β, but not IL-1⍺, has been implicated as the primary form of IL-1 involved in neural injury [40]. Consistent with these data, we observed increased circulating levels of IL-1β and elevated IL-1β mRNA expression in the periventricular white matter in the LPS+vehicle group compared to controls. By contrast, IL-1⍺ mRNA expression in the periventricular white matter did not differ between groups and IL-1⍺ was not detectible in plasma, most likely due to intracellular expression [41]. Critically, using a large animal translational model of perinatal infection/inflammation at term, the present study shows that IL-1β plays an important role in the pathophysiology of white matter inflammation and injury, and that targeted systemic inhibition can improve histological and functional outcomes.

Consistent with previous studies from our laboratory and others, LPS infusions were associated with a systemic inflammatory response as shown by elevated cytokine levels, systemic hypotension and tachycardia [7, 33, 42–44]. Repeated LPS infusions were associated with tolerance to subsequent doses (particularly following the final LPS infusion) indicating reprogramming of the innate immune system. In human and sheep monocytes, repeated LPS exposure is associated with decreased cytokine production and downregulation of the LPS receptor CD14 [45–47]. Consistent with these findings, in vivo studies in fetal sheep have shown that repeated LPS exposure is associated with attenuation of systemic inflammation [33, 44, 48].

In the present study, we used the commercially available IL-1Ra, Anakinra, to inhibit IL-1-mediated systemic and central nervous system inflammation in near-term fetal sheep. Anakinra is a recombinant non-glycosylated form of the human IL-1Ra and has been FDA approved for treatment of chronic inflammatory conditions in adults and children. It exerts its physiological effects by binding to the IL-1 receptor and neutralising the effects of IL-1 to prevent downstream inflammatory signalling [49]. It has a half-life of 4-6 h, weighs 17 kDa and can penetrate the blood-brain barrier in humans and sheep [30, 50]. Due to a lack of serial CSF sampling and the duration between the final dose of IL-1Ra and CSF collection (2 days), we were unable to determine whether IL-1Ra levels reached therapeutic concentrations in the brain. However, in adults, intravenous IL-1Ra administration was shown to cross the blood-brain barrier and achieve therapeutic concentrations within approximately 45 min [30].

To the best of our knowledge, the temporal profile of circulating cytokines has not been assessed in the setting of IL-1Ra and systemic inflammation in the near-term fetus. Infusion of IL-1Ra starting 1 h after LPS-induced inflammation led to a sustained reduction in circulating IL-6, from 6 h after the first LPS infusion, and reduced circulating IL-1β, TNF and IL-10 concentrations after the second LPS infusion. These data are consistent with in vitro and in vivo studies that reported inhibition of pro- and anti-inflammatory cytokines after IL-1Ra administration in adults with chronic inflammatory disease [51, 52], fetal sheep exposed to intra-amniotic LPS [53] and neonatal mice exposed to antenatal LPS and/or postnatal hyperoxia [54]. Collectively, these data demonstrate exogenous IL-1Ra can modulate systemic pro- and anti-inflammatory cytokine production in the fetus and neonate.

Elevated circulating levels of IL-1β are associated with impaired cerebral oxidative metabolism [20] and EEG suppression in neonates [55]. Similarly, in the present study, we observed suppression of EEG power and nuchal EMG activity (reflecting reduced neural activity and fetal movement, respectively) after the first LPS infusion, and sustained reductions in neural activity and foetal movement during the recovery period. The suppression of EEG power and foetal movement may reflect inhibition of synaptic activity due to increased local cytokine production and/or hypoxia. Indeed, suppression of EEG activity and foetal movement after the first LPS infusion was associated with mild reductions in arterial PaO_2_ and SaO_2_. Inflammation and cerebral hypotension/hypoperfusion can trigger active EEG suppression through release of inhibitory neuromodulators and neurosteroids [56–58]. Although in the present study systemic hypotension and EEG suppression were not associated with reduced carotid artery perfusion, there was an increase in circulating lactate concentration in LPS+vehicle and LPS+IL1Ra-treated groups after the first and second LPS infusions, suggesting impaired oxidative phosphorylation in response to LPS-induced inflammation at those times. These data are consistent with previous studies in preterm fetal and newborn sheep and raise the possibility that higher cerebral metabolic demand during fetal inflammation increases susceptibility to hypoxic-ischemic injury [7, 33, 59].

This concept is supported by studies in preterm and term neonates that linked antenatal/perinatal inflammation with disturbances in cerebral oxidative metabolism, as shown by increased cerebral oxygen consumption on near-infrared spectroscopy [60] and impaired cerebral oxidative metabolism on magnetic resonance spectroscopy [20]. By contrast, during the recovery period, when systemic oxygenation had normalised, suppression of EEG power and fetal movement in LPS-exposed fetuses was associated with increased brain tissue IL-1β immunoreactivity. Thus, passive anoxic depolarization and inflammation-induced synaptic inhibition may have modulated EEG activity in LPS-exposed foetuses.

In LPS+IL-1Ra-treated fetuses, we observed a reduction in the duration of EEG suppression and faster recovery of carotid artery perfusion after the first and second LPS infusions compared to the LPS+vehicle group, suggesting improved cerebral metabolism. Furthermore, arterial lactate concentration in the LPS+IL-1Ra group did not differ from controls but was higher in the LPS+vehicle group after the second LPS infusion, suggesting an intermediate improvement in oxidative phosphorylation with IL-1Ra treatment. During the recovery period, EEG power and foetal movement were improved in the LPS+IL-1Ra-treated group. Collectively, these data suggest that IL-1 plays an important role in mediating EEG suppression during foetal inflammation.

LPS-induced fetal inflammation was associated with increased numbers of total and activated microglia, in addition to increased caspase-3+ cells and IL-1β immunoreactivity, within the intragyral and periventricular white matter tracts. This was accompanied by reduced numbers of astrocytes and total (olig-2+) oligodendrocytes in the intragyral and periventricular white matter. The reduction in the numbers of astrocytes likely represents the acute phase of injury during neuroinflammation. For example, reduced astrocyte numbers were reported 48 h after hypoxia-ischemia in neonatal piglets and in mechanically ventilated newborn lambs [34, 61]. There was no apparent effect LPS-exposure or IL-1Ra treatment on myelination, as shown by no differences between groups in numbers of immature and mature oligodendrocytes expressing the CNPase protein, area fraction of CNPase staining, numbers of mature CC1+ oligodendrocytes or area fraction of MBP staining. These data are broadly consistent with previous studies in near-term fetal sheep that were exposed to LPS during a similar time-course [62]. This combination of reduced total (olig-2+) oligodendrocytes with no change in the numbers of immature and mature (CNPase+) or CC1+ mature oligodendrocytes or myelination suggests selective loss of late oligodendrocyte precursors in LPS-exposed near-term fetuses.

Similarly, we found no overt neuronal loss after LPS-exposure, as shown by no differences in cortical and striatal NeuN staining between the groups. The timing of white matter vulnerability in the near-term brain overlaps with late oligodendrocyte precursor cell proliferation [63]. These data suggest that late oligodendrocyte precursors in the near-term brain are relatively vulnerable to LPS-induced fetal neuroinflammation. Pathologically, these data are highly consistent with clinical findings of diffuse white matter injury in cases of term neonatal encephalopathy [9, 64].The combination of diffuse white matter gliosis with selective oligodendrocyte loss and relative sparing of the grey matter suggests that the pathological outcomes in the present study are comparable to a mild injury pattern in human neonates, consistent with mild neonatal encephalopathy [64, 65].

IL-1Ra treatment during LPS-induced fetal inflammation was associated with reduced induction of total and activated microglia, IL-1β immunoreactivity and caspase 3+ cells within the large white matter tracts but had no effect on astrocyte survival. The reduction in microgliosis was associated with increased total numbers of oligodendrocytes but had no effect on numbers of immature and mature oligodendrocytes (CNPase+ cells), mature oligodendrocytes (CC1+ cells) or myelination (CNPase and MBP immunoreactivity) and was associated with reduced olig-2/caspase 3+ co-labelling. Collectively, these data indicate that IL-1Ra was associated with improved survival of oligodendrocyte precursors. These data are consistent with evidence that systemic and/or locally produced IL-1β is involved in microglial infiltration and activation, and white matter injury [17, 66–69]. Furthermore, our data support a critical role for microglial activation in mediating acute oligodendrocyte loss during fetal inflammation [70] and indicate that targeted inhibition of IL-1β may be a viable therapeutic intervention.

Consistent with these observations, administration of IL-1Ra to neonatal rats exposed to LPS or LPS and hypoxia-ischemia was associated with reduced brain tissue IL-1β expression, reduced gliosis, improved myelination and improved motor and cognitive function [18, 71]. In the intragyral white matter tracts, we observed increased numbers of total (olig-2+) oligodendrocytes in LPS+IL-1Ra-treated fetuses compared to vehicle controls. Oligodendrocytes and their progenitor cells express IL-1 receptors [72, 73]. Binding of the IL-1 receptor on oligodendrocytes is a key extracellular signal for initiating oligodendrocyte apoptosis [74]. Further, IL-1β has been reported to promote oligodendrocyte cell death through glutamate excitotoxicity, most likely mediated by IL-1β-induced impairment of glutamate metabolism and/or uptake [74]. Consistent with these data, Leitner et al. showed IL-1Ra attenuated the inflammation-induced increase in NMDAR1 expression and reduced nNOS activation in primary cortical neuronal cultures from preterm mice exposed to intrauterine inflammation [75]. Thus, the increased number of total (olig-2+) oligodendrocytes in the LPS+IL-1Ra group compared to controls was likely mediated by local anti-excitotoxic and anti-apoptotic effects of IL-1Ra treatment. Furthermore, upregulation of caspase-3 has also been linked to microglial and lymphocyte proliferation/activation, cell differentiation and autophagy [76–78]. In line with these observations, the reduction in caspase-3 staining observed after IL-1Ra treatment was not associated with improved astrocyte survival, indicating IL-1Ra treatment had a selective anti-apoptotic effect on oligodendrocytes and may have impacted on other pathophysiological pathways induced by caspase-3 expression.

In the LPS+IL-1Ra-treated group, we observed faster restoration of carotid artery perfusion to baseline levels after LPS infusions compared to the LPS+vehicle group. However, IL-1Ra treatment was associated with a similar magnitude and duration of hypotension and tachycardia after LPS infusions compared to the LPS+vehicle group. These data indicate that IL-1Ra did not worsen or improve the inflammation-induced cardiovascular effects of fetal LPS administration and suggests that the improved recovery of carotid artery perfusion in the IL-1Ra-treated group was mediated by a local anti-inflammatory effect on the cerebral vasculature and/or tissue. Supporting these data, intracisternal injection of IL-1β in adult dogs was associated with a dose dependant increase in basilar artery perfusion and vasodilation, without affecting systemic blood pressure or heart rate. Furthermore, IL-1Ra treatment reduced the IL-1β-mediated increase in cerebral artery vasodilation and perfusion, which was likely mediated by IL-1β-induced prostaglandin production [79]. By contrast, in preterm foetal sheep, systemic TNF blockade was associated with inhibition of systemic hypotension and tachycardia during LPS-induced inflammation [33]. Collectively, these data suggest that IL-1β has targeted effects on the cerebral vasculature whereas other cytokines, including TNF, play a greater role in modulating the cardiovascular adaptations to systemic inflammation in the fetus.

One of the key translational considerations for potential neuroprotectants is when to treat [80–82]. In the present study, we started IL-1Ra infusions 1 h after infusing LPS. The rationale was to establish proof-of-concept that systemic IL-1Ra started after early-onset foetal inflammation can alleviate neuroinflammation and injury. However, it is important to appreciate that in current practice it is unlikely that inflammation (infectious or sterile) can be detected and treated as soon as it begins. Thus, the present study supports further investigation to determine the window of opportunity of IL-1Ra for treating inflammation-induced brain injury.

## Conclusions

In conclusion, IL-1Ra administration to near-term fetal sheep during LPS-induced inflammation prevented microgliosis, reduced circulating cytokines and white matter IL-1β expression and improved survival of oligodendrocyte progenitors and EEG recovery 4 days after starting LPS infusions. These data suggest a possible anti-inflammatory and neuroprotective role for IL-1β inhibition in infants exposed to infection/inflammation during the perinatal period. Based on these data, further translational studies are needed to evaluate the effects of IL-1β inhibition on long-term myelination, the optimal dosing regimen, and the efficacy of delayed administration to assess the potential for IL-1Ra to improve outcomes after birth.

## Supplementary Information

**Additional file 1 **: **Supplementary Figure 1**. Negative controls. Representative photomicrographs of LPS+vehicle sections that had the target antibodies (oligodendrocyte transcriptase factor-2, olig-2, green and cleaved caspase-3, red) omitted. Sections were incubated in 1:500 donkey anti-mouse-Alexa Fluor 488 and 1:500 donkey anti-mouse-Alexa Fluor 647 for 1 h at room temperature. Left panel shows immunofluorescent staining of 4′,6-diamidino-2-phenylindole (DAPI, showing cell nuclei, blue). Middle panel shows sections without the target antibody did not display non-specific staining. Right panel shows the merged image. Scale bar = 50 μm.

## Data Availability

The datasets used during the current study are available from the corresponding author upon reasonable request.

## Abbreviations

CaBF: Carotid arterial blood flow
CaVC: Carotid arterial vascular conductance
CNPase: 2′, -3′-Cyclic-nuceotide 3′-phosphodiesterase
EEG: Electroencephalogram
FHR: Fetal heart rate
GFAP: Glial fibrillary acidic protein
Iba-1: Ionised calcium-binding adapter molecule-1
IGWM: Intragyral white matter
IL: Interleukin
LPS: Lipopolysaccharide
MAP: Mean arterial blood pressure
Olig-2: Oligodendrocyte transcription factor-2
PBS: Phosphate-buffered saline
PVWM: Periventricular white matter
TNF: Tumour necrosis factor
