# Supplementary material for: Interleukin-1 blockade attenuates white matter inflammation and oligodendrocyte loss after progressive systemic lipopolysaccharide exposure in near-term fetal sheep
Source: J Neuroinflammation. 2021 Aug 31;18:189. doi: 10.1186/s12974-021-02238-4 (PMC8408978; doi:10.1186/s12974-021-02238-4)
Supplement: Supplementary file 1 — Additional file 1 : Supplementary Figure 1. Negative controls. Representative photomicrographs of LPS+vehicle sections that had the target antibodies (oligodendrocyte transcriptase factor-2, olig-2, green and cleaved caspase-3, red) omitted. Sections were incubated in 1:500 donkey anti-mouse-Alexa Fluor 488 and 1:500 donkey anti-mouse-Alexa Fluor 647 for 1 h at room temperature. Left panel shows immunofluorescent staining of 4′,6-diamidino-2-phenylindole (DAPI, showing cell nuclei, blue). Middle panel shows sections without the target antibody did not display non-specific staining. Right panel shows the merged image. Scale bar = 50 μm. [file 12974_2021_2238_MOESM1_ESM.docx]

Supplementary figure 1

Supplementary figure 1 legend

Negative controls. Representative photomicrographs of LPS+vehicle sections that had the target antibodies (oligodendrocyte transcriptase factor-2, olig-2, green and cleaved caspase-3, red) omitted. Sections were incubated in 1:500 donkey anti-mouse-Alexa Fluor 488 and 1:500 donkey anti-mouse-Alexa Fluor 647 for 1 h at room temperature. Left panel shows immunofluorescent staining of 4′,6-diamidino-2-phenylindole (DAPI, showing cell nuclei, blue). Middle panel shows sections without the target antibody did not display non-specific staining. Right panel shows the merged image. Scale bar = 50 µm.
